# The Effects of Pyridaben Pesticide on the Histomorphometric, Hormonal Alternations and Reproductive Functions of BALB/c Mice 

**Published:** 2013-10

**Authors:** Ghodrat Ebadi Manas, Shapour Hasanzadeh, Kazem Parivar

**Affiliations:** 1 Department of Biology, Science and Research Branch, Islamic Azad University, Tehran, Iran; 2 Department of Basic Veterinary Sciences, Histology and Embryology Sections, Faculty of Veterinary Medicine, Urmia University, Urmia, Iran

**Keywords:** Hormones, Male mouse, Pyridaben, Reproduction, ROS, NOS

## Abstract

***Objective(s):*** The adverse effects of pyridaben on reproductive system in male animals are not well established. This study was designed to elucidate how pyridaben can effects the histomorphometric, hormonal alternations and reproductive functions of BALB/c mice.

***Materials and Methods***: For this study, 80 adult and apparently healthy male BALB/c mice were divided into three groups *Viz*, control, test group 1 and test group 2. Test groups 1 and 2 were received the toxin at doses of 53 mg/kg. BW, and 212 mg/kg. BW, respectively. The experiment period for both groups was 10, 25 and 45 days.

***Results***: The levels of FSH, LH and testosterone were significantly (*P<*0.05) decreased on the dose and time dependant means. The levels of the ROS and NOS were significantly (*P<*0.05) increased in all test groups. The percent body weight gains significantly (*P<*0.05) reduced, whereas weights significantly (*P<*0.05) increased in test groups in a dose and time dependant manner. The histomorphometric and stereologic findings, including diameters of somniferous tubules, thickness of somniferous tubules epithelium, the leydig's cell distribution, TDI, SI, RI revealed that, all these parameters are also significantly (*P<*0.05) reduces in test groups in a dose and time dependant manner.

C***onclusion***: Pyridaben causes histomorphometric and stereologic changes in testis, as well as hormonal and reproductive functional alternations in BALB/c mice.

## Introduction

Pyridaben, Code No. NC-129, NCI-129, Chemical name: 2-tert-butyl-5-(4-tert-butylbenzylthio)-4-chloropyridazinone-3(2h)-one, a pyridazinone derivative which is the most recently introduced acaricides for the control of Tetranychus urtica in the world (1, 2).

Pyridaben has been studied for its acute toxicity in different species (3, 4). The inhabitation of complex I by pyridaben directs mtNOS to loose it’s NO producing activity and to become an O_2_^-^ source (4-6). 

Pyridaben is two times more potent than rotenone in inhibiting mitochondrial respiration, and is a competitive inhibitor of DHR (dihydrorotenone) binding, (5, 7, 8). Pyridaben causes significantly greater oxidative damage than a similar dose of rotenone as measured by protein carbonyl. Partial inhibition of complex I by pyridaben is sufficient to produce significant increase in reactive oxygen species (ROS) production (9-16). Pyridaben has not been fully evaluated for its possible immunotoxic effects, but it has been demonstrated that, it is a potent inducer of apoptosis in ST486 and in EW36 cells in combination with heat stress (17). It encompasses anticancer action (18).

The production of reactive oxidative oxygen species in the male reproductive system due to their high toxic effects on the quality and potential of action of sperms is an important concept in andrology (19). Although small amount of ROS is necessary for sperm potentiality in fertilization, acromosomal reaction, motility and capacitation (20*)*, some studies have been revealed that 25-45% of infertile men showing high levels of ROS in their semens (21, 22). The spermatozoa are showing special sensitivity to oxidative stresses damages, because their plasma membrane exhibiting profound amounts of polyunsaturated fatty acids (PUFAs), and in their cytoplasm small amount of inhibitory enzymes is present (23, 24). 

Apart from this, the antioxidant enzymes present inside the cells are not able to protect surrounding plasma membranes of acrosome and tails of spermatozoa, thus, in order to reinforce their limited intracellular antioxidant system, cells get help from protective mechanisms of semen plasma (25-27). Previous studies have revealed that there is a direct relationship between sperm quality and increased ROS production (28).

Pyridaben inhibits NADH-ubiquinone oxidoreductase activity, leading to the level of induced ornithine decarboxylase activity which in turn causes the antiproliferative effect, and since the ROS causes mitochondrial membrane damage, damaged mitochondrial membrane exaggerates ROS production (29*)*. ROS invade polyunsaturated fatty acids located at cell membrane, which in turn leads to lipid peroxidation (30*, *31*)*, and finally free radicals liberation starts *(*32*)*. One of the lipid peroxidation by-products is malondialdehyde (MDA). This by-product is used in different biochemical assays for detection of peroxidative damages (33*)*. Increased ROS production leads to reduction in sperm motility *(*34*)*. Increased ROS level leads to sperm DNA damage and this damage causes structural changes in DNA bases such as removal of DNA bases in some loci, removal or DNA bases frame shift, formation of unnatural bonds between two DNA strands, chromosomal recombinant (35). Apart from these, oxidative stress enhances breaks in one DNA strand or both of them *(*36*)*. Increase in the amounts of ROS also is able to cause different forms of gene mutations such as point mutation and multiforms, which lead to reduction in sperm quality *(*37*)*. If the amounts of damages to DNA are negligible, sperm or even oocyte will be able to repair DNA *(*38*), *but in cases the damages are more pronounced the apoptosis and fragmentation of embryonic cells nuclei which are obtained proceeding such sperms and or oocytes fertilization are more likely to take place. Reductions in fertility and quality of embryo are in direct proportional relation with the size of DNAs damages *(*39*)*. DNA damage can also take place in Y chromosome and may lead to gene deletions in offsprings and finally lead to infertility *(*36*)*. The elevated levels of ROS can also cause disturbances in inner and outer mitochondrial membranes, consequently cytochrome c seepage could take place, and activation of caspase enzymes causes apoptosis (40*)*. Previous studies revealed that, reaction of ROS with mitochondria -induces liberation of apoptosis inducing factor (AIF) which directly reacts with DNA and causes DNA fragmentation *(*41*)*. Another study revealed that there is a direct relationship between ROS-induced damage to the sperms and elevated levels of cytochrome c and caspases 3 and 9 (42). As the deleterious effects of pyridaben on histomorphometric, hormonal alternations and reproductive functions of male mammals have not been studied, this is the first investigation from this aspect. 

## Materials and Methods

For this study, 80 adult and apparently healthy male BALB/c mice with body weight of 20±5.0 g were used. Before commencing the experiment, animals were left without any treatment for seven days for adaptation. Animals were fed on standard mice pellet and with tap water *ad libitum*, 12 hr light and 12 hr dark, temperature 20-23°C, conditions were strictly monitored.

***Animal groups***

Animals were divided into *viz *(3 groups), control, test group 1 and test group 2. The control group comprised of 20 mice, but the other groups comprised of 30 mice. The living conditions were equal for all groups, but experimental groups received pyridaben as described below.

***Experiment***

Mice in test group 1(treated with low dose of pyridaben) received pyridaben (53 mg/kg. BW), dissolved in distilled water and fed on orally (by gavages) for 45 consecutive days. Mice in test group 2 (treated with high dose of pyridaben) received pyridaben (212mg/kg BW, dissolved in distilled water), orally (by gavages) for 45 consecutive days. Mice in control group received the same volume of distilled water.

***Specimens***

In each stage, the body weights of mice were recorded, and then the animals sacrificed by advocated human means. Immediately after the sacrifice of animals all the blood present in their circulation was aspirated from the heart by special needle. Collecting the specimens was carried out in all groups on 10^th^, 25^th^ and 45^th^ day of experiment. In each occasion 10 mice from each group were sacrificed for acquiring specimens. Then, their abdominal region was cut opened and their testes along with epididymides were taken out and fixed in formalin saline solution. The other testis was stored in deep freeze for further biochemical analyses.

***Tissue preparation***

The formalin fixed testes were processed through routine paraffin embedding, cut at 6 µm and stained with Hematoxilin – Eosine and Weigert's - Iron techniques. All the morphometric parameters including; diameters of semniferous tubules (SFTs), height of SFTs, diameters of tunica albuginea testis, and the thicknesses of interstitial tissues were recorded.

***Cytomorphometry***

The distribution of leydig’s cells as well as lymphocytes in mm^2 ^of testicular tissue, the different testicular indices of spermatogenesis including RI (repopulations index), TDI (tubular differentiation index), and SI (sperm index) were recorded.

***Hormonal assays***

The hormonal assays including LH, FSH (Pishtaz teb kits – Zaman Diagnostics Tehran, Iran), were carried out on blood serum by ELISA techniques. Testosterone assay was carried out on blood serum by electrochemiluminescence techniques (Roshd kits: 05200067 190).

***Assessment of total antioxidant capacity (TAOC)***

The testicular tissues were processed for the evaluation of NOS, ROS, FRAP, and Thiol. To determine the effect of pyridaben on oxidative stress, testicular antioxidant capacity (TAOC) was measured. The assay is based on the assessment of ferric reduction antioxidant power (FRAP) assay (43). Briefly, at low pH which was achieved by using acetate buffer (300mM, pH 3.6), reduction of Fe^III ^– TPTZ complex to the ferrous form produces an intensive blue color that could be measured at 593 nm. Aqueous solution of Fe^II ^(FeSO_4._7H_2_O) and appropriate concentration of freshly prepared ascorbic acid were used as blank and standard solutions, respectively. The TAOC was expressed as mM per mg protein of the samples. The protein content of the samples was measured according to Lowry method (44).

## Results

***Histomorphology***

**Figure 1:**
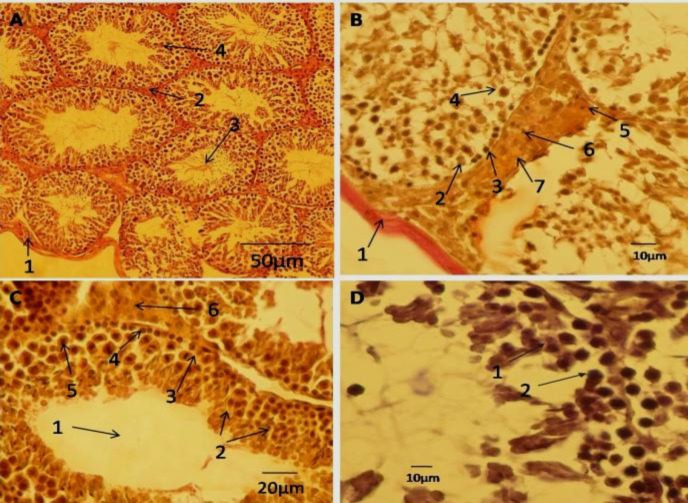
A– Cross section of testis belonging to control male mice, 1- tunica albuginea; 2-interstitial tissue; 3- luminal space of somniferous tubule containing spermatozoa; 4-spermatids, Weigert´s stain. B - cross section of testis belonging to high dose pyridaben treated male mice, 1- tunica albuginea; 2- type A spermatogonium; 3- type B spermatogonium, 4- primary spermatocyte, 5- leydig's cell, 6- lymphocyte ; 7- area of interstitial tissue, Weigert´s stain. C- cross section of testis belonging to low dose pyridaben treated male mice, 1- the free spermatozoa in lumen of semniferous tubule; 2- spermatids; 3- type A spermatogonium; 4- type B spermatogonium; 5- primary spermatocyte; 6- leydig's cells , Weigert´s sain. D- cross section of testis belonging to high dose pyridaben treated male mice with negative tubal differentiation index (TDI); 1- primary spermatocyte; 2-reduced germinal epithelium of somniferous tubule into two layers (TDI negative ), H&E stain

In male rats from control group, the microarchitecture and morphologic appearance of the testicular interstitial tissue, including tunica albuginea, leydig's cells and spermatogenic cell series of somniferous tubules were normal (Figure 1-A).

In male rats treated with low dose of pyridaben, alterations in the microarchitecture and morphologic appearance of the testicular interstitial tissue, including tunica albuginea, leydig’s cells and spermatogenic cell series of semniferous tubules were obvious. In this group round spermatids were observed, but spermatozoa not seen in the luminal provinces of somniferous tubules (Figure 1-C).

In rats treated with high dose of pyridaben, profound reduction in populations of all types of the spermatogenic cell series including spermatogonia, spermatocytes, round and elongated spermatids was obsereved, but the spermatozoa were absolutely absent. In the interstitial tissue, the integrity and population of leydig's were reduced; however, infiltration of lymphocytes was profound (Figure 1-B). 

In rats exposed to high dose of pyridaben for a period of 45 days, the epithelia of semniferous tubules were reduced to below 4 cell layers (TDI negative) ( Figure 1-D). 

***Histomorphometry and Stereology***

**Figure 2.A:**
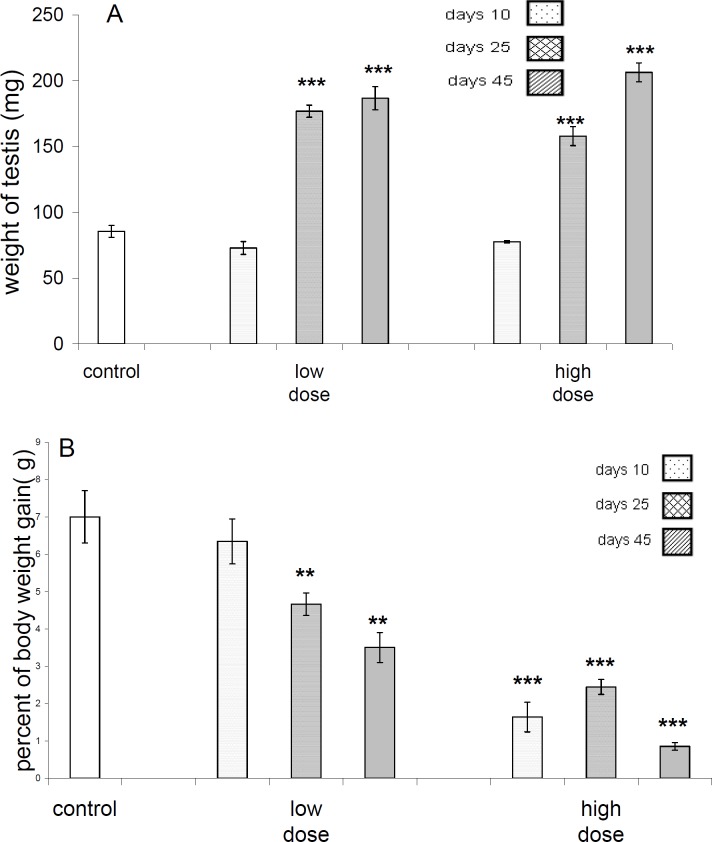
Weight of testes in control and different test groups. The weight of testes in low dose as well as high dose groups at 25 and 45 days after receive of pyridaben showing highly significant (*P<*0.001) increase. B-Percent of weight gains in control and different test groups. In low dose group at 25 and 45 days after treatment with pyridaben significant (*P<*0.001) decrease in percent of body weight gain are evident, but in high dose group in 10, 25 and 45 days after treatment high significant (*P<*0.001) decrease in this parameter are evident

Data analyses of weight gain percentages between test and control groups revealed that, after 45 days of exposure to the pyridaben (212 mg/kg.BW/days) significant difference (*P*<0.05) is attains, but there was no difference(*P*>0.05) between control with other test groups concerning this parameter (Figure 2).There was no significant difference (*P*>0.05) between the body weights of control and both of test groups after 10 days, but there was a significant difference between test groups and control after 25 and 45 days of exposure to pyridaben (Figure 2).

**Figure 3:**
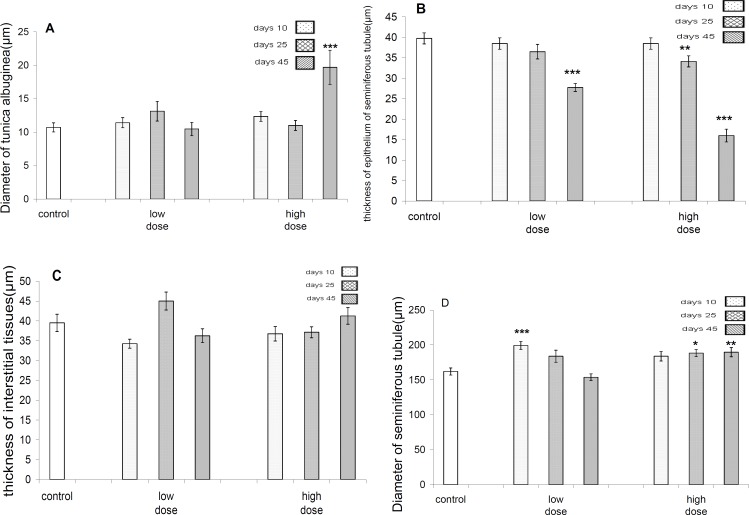
. A-Diameter of tunica albugina in control and different test and groups. There are no evident of differences in low dose group in comparison to control group, but in high dose group there is highly significant (*P<*0.001) increase in diameter of tunica albugina only after 45 days of pyridaben obtain. B- Thicknesses of epithelium of seminiferous tubules in control and different test groups. The significant (*P<*0.01) decrease in this parameter is evident in low dose group at 45 days, but in high dose group the decrease in this parameter were evident at 25 and 45 days. C- Thicknesses of testicular interstitial tissue not revealing differences in between test and control groups. D- Diameter of seminiferous tubules in test and control groups in low dose group a highly significant increase in this parameter is evident only at 10 days treatment ,but in high dose group this increase is evident at 25 and 45 days after exposure to pyridaben

There was significant difference (*P*<0.001) regarding tunica alboginea thicknesses between control and high dose group after 45 days. However, there was no significant difference (*P*>0.05) between control and other test groups after 25 and 45 days of exposure (Figure 3).

There was a significant difference (*P*<0.001) in diameters of seminiferous tubules between control and low dose-treated group after 10 days of exposure, but there was not a significant difference between control and low dose-treated groups after 25 and 45 days and there was significant difference between control and high dose-treated group after 25 and 45 days of exposure, but there was not a significant difference following 10 days of exposure (Figure 3).

Data analyses regarding diameters of seminiferus tubules epithelium revealed that there is a significant (*P*<0.001) difference between control and both of the test groups only after 45 days of exposure (Figure 3).

There was not a significant difference in thicknesses of interstitial tissue between control and all test groups (Figure 3).

**Figure 4.**
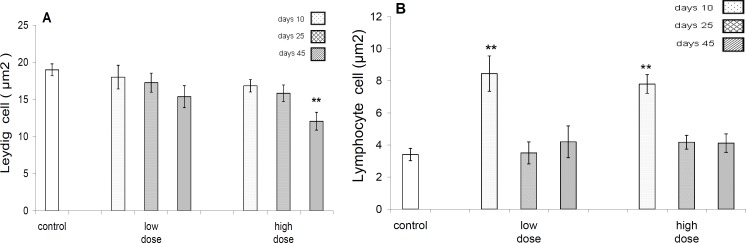
A -The mean distribution of leydig^٬^s cell (µm^2^) of testicular tissue in control and different test groups .A significant (*P<*0.01) decrease in this parameter is evident only in high dose group after 45 days exposure to pyridaben. B- The mean distribution of lymphocyte distribution (µm^2^) in testicular tissues of control and different test groups .The significant (*P<*0.01) increase in distribution of their cells evident at 10 days in both the low and high dose groups

There was significant difference (*P*<0.001) in leydig’s cells distribution in mm^2^ of testicular interstitial tissue between control and high dose-treated group after 45 days of exposure, but there was no significant difference between control and other test groups (*P* >0.05) (Figure 4).

There was a significant difference in lymphocytes distribution in mm^2^ of testicular interstitial tissue between controls with both test groups only after 10 days of exposure (Figure 4).

**Figure 5:**
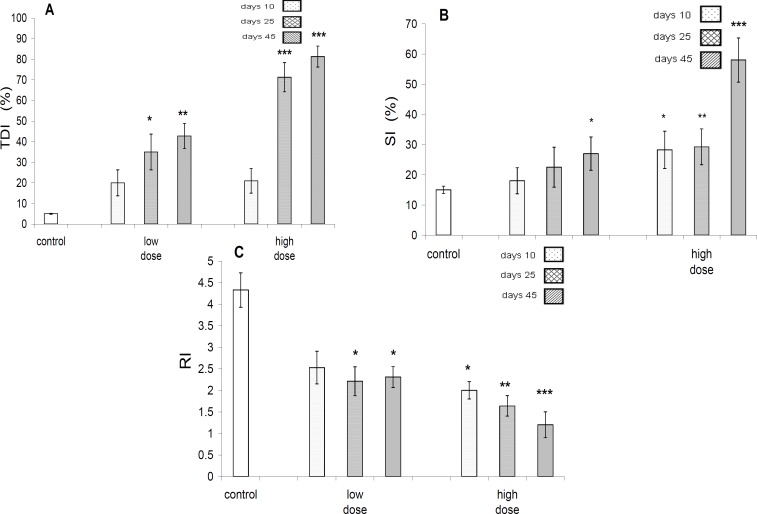
A-TDI (%), in control and different test groups. The TDI (%) increase is significant at 25, but highly significant (*P<*0.01) at 45 days in low dose group, whereas very highly significant (*P<*0.001) at 25 and 45 days in high dose group in comparison to control group. B- SI (%) in control and test groups. A significant (*P<*0.05) increase in this parameter is evident in 45 days after exposure to low dose of pyridaben, but in high dose group this was significant (*P<*0.05) at (10 days), highly significant (*P<*0.01) at 25 days and very highly significant at 45 days. C- RI in control and different test groups. Significant (*P<*0.05) decrease in this parameter are evident at 25 and 45 days of low dose group, but at 10 days, (*P<*0.05) 25 days, (*P<*0.01), and 45 days (*P<*0.001) of high dose groups

Data analyses concerning TDI(testicular differential index), revealed that there is no significant difference between control and test groups after 10 days of exposure, but there is a significant(*P*<0.001) difference between control and other test groups after 25 and 45 days of exposure (Figure 5).

Data analyses concerning SI(sperm index) revealed that, there is not a significant (*P*>0.05) difference between control and low dose-treated group after 10 and 25 days ,but there was a significant difference between control and low dose-treated test group after 45 days. In addition, there was a significant difference between control and high dose-treated group in 10, 25 and 45 days (Figure 5).

Analyses of RI (repopulation index) data revealed that, there is not a significant difference (*P*>0.05) between control and low dose-treated group after 10 days of exposure, but there was significant difference between control and other test groups (Figure 5).

***Hormonal analyses***

**Figure 6:**
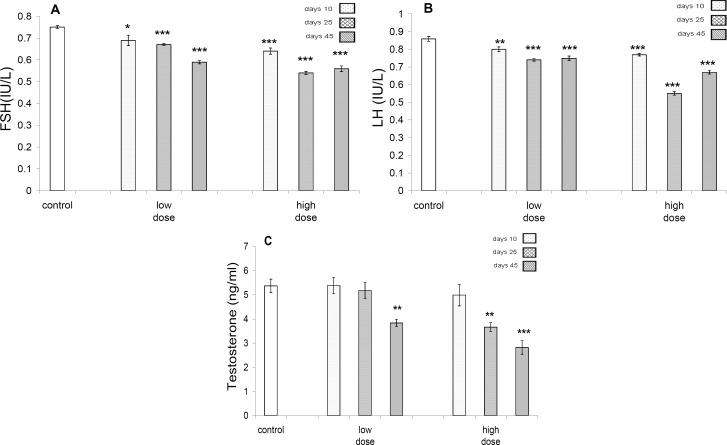
A- FSH profile in control and different test groups. The significant decrease are evident in 10 days (*P<*0.05), 25 days (*P<*0.001) and 45 days (*P<*0.001) in low dose group, but in high dose groups the reduction in profile of this hormone in 10, 25, 45 days after exposure are highly significant (*P<*0.001). B- LH profile in control and different test groups. In both of low dose and high dose groups highly significant (*P<*0.001) decrease in profile of this hormone is evident. C-Profile of testosterone in control and different test groups in low dose group significant decrease in profile of this hormone is significant (*P<*0.01) in 45 days, but in high dose group it is significant (*P<*0.01) at 25 days, but highly significant (*P<*0.001) in 45 days

Significant decreases were seen in FSH level following 10 days (*P*<0.05), 25 days (*P*<0.001) and 45 days (*P*<0.001) in low dose-treated group, but in high dose-treated groups the reduction in profile of this hormone, 10, 25, and 45 days after exposure was significant (*P*<0.001). In both low and high dose-treated groups, significant (*P*<0.001) decrease in profile of LH was evident (Figure 6). Profile of testosterone in control and different test groups in low dose-treated group revealed significant difference (*P*<0.01) after 45 days exposure, but in high dose-treated group, it was significant (*P*<0.01) after 25 days of exposure, whereas it was significant (*P*<0.001) after 45 days of exposure (Figure 6).

***TTM (Total Thiol Molecules)***

**Figure 7:**
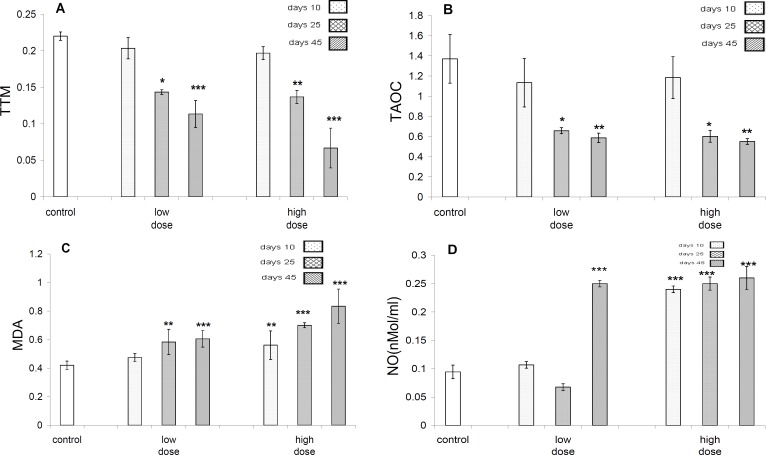
A- Profile TTM (total thiol molecules. In low dose test groups, the amount of TTM reduced at 25 days (*P<*0.05), and at 45 days (*P<*0.01), but in high dose group in 25 days (*P<0.01*), and at 45 days (*P<*0.001), on comparison to control group. B- Profiles of total antioxidant capacity (TAOC) in control and test group. In both of test groups, at 25 days (*P*<0.05) and 45 days (*P<*0.01) significant reduction are evident. C-The profiles of malondealdehyde (MDA) in control and test groups. In the low dose test group significant (*P<*0.01) increase in 25 days and highly significant (*P<*0.001) increase at 45 days are evident, but in high dose group, significant increase at 25 (*P<*0.001) and 45 days (*P<*0.001) are apparent. D- Profiles of NO in control and test groups. In low dose group highly significant increase in 45 days (*P<*0.001) only, but in high dose group in 10, 25 and 45 days highly (*P<*0.001) increase are apparent

Data analyses revealed that there is no significant difference in the amounts of TTM between all groups after 10 days of exposure to pyridaben, whereas, after 25 days of exposure there was significant (Figure 7) difference between control and test groups regarding the amount of TTM, whereas there was not any difference between test groups. On the other hand, after 45 days of exposure there was significant difference between control and test groups from the aspect of the amount of TTM (Figure 7).

***TAOC (Total antioxidant capacity)***

In both test groups, after 25 days (*P*<0.05) and 45 days (*P*<0.01) of exposure, significant reduction was evident in the amounts of TAOC (Figure 7).

***MAD (malondealdehyde)***

Statistical analysis on the profile of malondialdehyde (MDA) in control and test groups revealed that in the low dose-treated test group significant (*P*<0.01) increase following 25 days of exposure and highly significant (*P*<0.001) increase at 45 days exposure are evident, but in high dose-treated group, significant increase after 25 (*P*<0.001) and 45 days (*P*<0.001) are evident (Figure 7).

***NO (Nitric oxide)***

The profiles of NO in control and test groups in low dose-treated group revealed highly significant (*P*<0.001) increase only after 45 days of exposure, but in the high dose-treated group after 10 days (*P*<0.001), 25 days (*P*<0.001) and 45 days of exposures (*P*<0.001), highly significant increase was obvious (Figure 7). 

## Discussion

Pyridaben, a pyridazinone derivative, is an acaricide and insecticide (45). The other compounds commonly used as pesticide are atrazine, benomyl, carbaryl, carbofuran, chlorpyrifos, endosulfan, dibromochloropropane(DBCP), 2, 4- dichlrophenoxy acetic acid, dimethoate, dioxin, hexachlorocyclohexane(HCH), lindane, malathione, mancozeb, methoxychlor, methyl parathion, organophosphate and pyrethyroid. The reproductive toxic effects of these compounds are widely studied (46). At present, Pyridaben is widely used as miticide and insecticide all over the world, although this compound is toxic for different biological systems (18, 45, and 47) but its noxious effects on male and female reproductive systems are inadequately known. For this reason, we designed this investigation to find out the deleterious effects of this compound on different parameters of male mice reproductive system, including body weight, testis weight, the histomorphologic, histometric and stereological evidences (including diameters of tunica albuginea, semniferous tubules, testicular interstitial tissue, Leydig´s cells and lymphocyte distribution on testicular tissue, TDI, SI, and RI). Besides, the hormonal assays (including FSH, LH and testosterone) and evaluation of ROS and NOS biomarkers were carried out.

According to the results of our study, pyridaben time and dose dependently causes reduction in percentage of body weight gain in male mice. According to previous studies, body weight decreases in methyl parathion treated rats (48). Results of our study revealed that increase in testes weight occurs in a dose and time dependant pattern. The increase in testes weight is highly likely due to the edemas of testes caused by the pyridaben. Our morphological observations confirmed this phenomenon. The edema of testes is reported by Joshi *et al* (49) and Ngoula *et al* (50) in male rat by chloropyrifos and pirimiphos –methyl, respectively. It was shown that, a carbamate pesticide (2-isopropoxy-phenyl-N-methylcarbamate) which administered to adult male Wistar rats for 90 successive days leads to a concentration-dependent increase in relative weights of testis and epididymis and a decrease in sperm density, serum and intratesticular total cholesterol concentrations, and intratesticular total proteins (50). 

According to results of this study, pyridaben results in an increase in thickness of the tunica albugina of the testes in test groups in a concentration and time dependent pattern. According to Kulshrestha and Neelam (51), carbaryl and endosulfan pesticides cause increases in ovarian tunica albugina thickness in fresh water teleost *Chana straitus*, following the exposure for a period of 2-30 days during the spawning season. 

Our studies on the effects of pyridaben on the thickness of the seminiferous epithelium revealed that, this parameter decreases in a concentration and time dependent pattern. There were sloughing and disorganization of spermatogenic cells with their exfoliation in luminae of seminiferous tubules. It was demonstrated that degenerative changes in the seminiferous tubules occur in rats receiving malathion (27mg/kg/day for 4 weeks) (52). Thus, the reduction in thickness of the somniferous tubules epithelium is due to the degenerative effects of the pesticides.

There were no considerable changes in the testicular interstitial tissues in test groups treated by pyridaben. Also, malation does not lead to changes in the interstitial tissue of the testes (52).

Our evaluation on the distribution of the Leydig´s cells in the interstitial tissue of the testes revealed that significant decrease in the population of these cells occurs only after 45 days administration of the pyridaben at high dose (212 mg/kg). Ngoula *et al* (50), stated that, pirimiphos-methyl, an organophosphothioate pesticide, causes rarefaction of Leydig's cells.

Our assessments on the distribution of the lymphocytes in the interstitial tissue of the testes revealed that significant increase in the population of these cells occurs after 10 days of daily exposure to the pyridaben at low and high doses but not after 25 and 45 days. It appears more likely that, by first contact with the toxin, a shock like increase in the lymphocytes happens and consequently adjustment and adaptation occurs, that is why in both doses after 25 and 45 days of treatment population of lymphocyte distribution are the same as in control group.

The results of this study revealed that, pyridaben results in an increase in TDI percentage in a dose and time dependant mode. It is to be noticed that, when the TDI percentage increases, it indicates that the epithelial layer is undergoing degeneration and thinning. The previous studies reported that, dimethoate (53) causes dose related testicular damage characterized by moderate to severe seminiferous tubule degeneration as sloughing, atrophy, germ cell degeneration and partial arrest of spermatogenesis. Farag *et al* (54) reported that, dimethoate causes adverse effects on reproductive performance of male mice, including sperm viability, motility and density. Such changes are reported by Joshi *et al* (49), which were caused by Chlorpyrifos.

We noticed that the RI decreases in a dose and time dependant manner. This indicates that, the ratio of spermatogonia type B to the spermatogonia type A is reducing by the pyridaben, thus this toxin causes decrease in spermatogonia type B population. High dose of 2-bromopropane decreases spermatogenesis by adversely affecting spermatogonia followed by depletion of spermatocytes, spermatids, and spermatozoa with subsequent testicular atrophy (55). 

 The SI percentage, i.e., percentage of seminiferous tubules without sperms, was increased by pyridaben as the dose and time increase. Dimethoate was shown to be able to decrease sperm viability, motility and density (56).

Our results indicated that, pyridaben causes decrease in the profiles of FSH, LH and testosterone levels based on concentration and exposure duration of the received toxin. It was reported that carbamate poisoning in male rats causes a significant decrease in the level of testosterone, whereas the levels of the FSH and LH increase (57). Quinalphos, a commonly used organophosphorus insecticide caused a reduction in plasma levels of testosterone, FSH, and LH hormones (58).

According to our results, the malondialdehyde (MDA) and NO levels are increased based on the dose and exposure duration of the toxin. The decrease in antioxidant enzymes by induction of ROS production causes increase in the levels of MDA and NO by pyridaben toxin. The total thiol molecules (TTM), and total antioxidant capacity (TAOC), are decreased in our experiment. The activities of antioxidant enzymes such as superoxide dismutase, catalase, glutathione reductase and glutathione peroxidase decrease in testes proceeding methoxychlor pesticide intoxication male rats (59). 

## Conclusion

According to the results of this stud we conclude that, pyridaben causes histomorphometric changes of testis, as well as hormonal alternations and increase in the levels of ROS and NOS along with changes in reproductive functional in BALB/c mice.
